# Noise Reduction in Brain CT: A Comparative Study of Deep Learning and Hybrid Iterative Reconstruction Using Multiple Parameters

**DOI:** 10.3390/tomography10120147

**Published:** 2024-12-18

**Authors:** Yusuke Inoue, Hiroyasu Itoh, Hirofumi Hata, Hiroki Miyatake, Kohei Mitsui, Shunichi Uehara, Chisaki Masuda

**Affiliations:** 1 Department of Diagnostic Radiology, Kitasato University School of Medicine, Sagamihara 252-0374, Japan; 2 Department of Radiology, Kitasato University Hospital, Sagamihara 252-0375, Japan

**Keywords:** computed tomography, brain, deep learning reconstruction, hybrid iterative reconstruction, image noise

## Abstract

Objectives: We evaluated the noise reduction effects of deep learning reconstruction (DLR) and hybrid iterative reconstruction (HIR) in brain computed tomography (CT). Methods: CT images of a 16 cm dosimetry phantom, a head phantom, and the brains of 11 patients were reconstructed using filtered backprojection (FBP) and various levels of DLR and HIR. The slice thickness was 5, 2.5, 1.25, and 0.625 mm. Phantom imaging was also conducted at various tube currents. The noise reduction ratio was calculated using FBP as the reference. For patient imaging, overall image quality was visually compared between DLR and HIR images that exhibited similar noise reduction ratios. Results: The noise reduction ratio increased with increasing levels of DLR and HIR in phantom and patient imaging. For DLR, noise reduction was more pronounced with decreasing slice thickness, while such thickness dependence was less evident for HIR. Although the noise reduction effects of DLR were similar between the head phantom and patients, they differed for the dosimetry phantom. Variations between imaging objects were small for HIR. The noise reduction ratio was low at low tube currents for the dosimetry phantom using DLR; otherwise, the influence of the tube current was small. In terms of visual image quality, DLR outperformed HIR in 1.25 mm thick images but not in thicker images. Conclusions: The degree of noise reduction using DLR depends on the slice thickness, tube current, and imaging object in addition to the level of DLR, which should be considered in the clinical use of DLR. DLR may be particularly beneficial for thin-slice imaging.

## 1. Introduction

Computed tomography (CT) reveals the anatomy and pathology of patients noninvasively and plays an essential role in modern medicine. However, the potential detriments of radiation exposure are a significant concern in CT clinical practice. Brain CT is frequently used in various situations such as stroke and trauma. Although the radiation dose is generally lower in brain CT than in body CT, epidemiological studies have demonstrated an increased incidence of brain tumors in children who underwent brain CT [1,2,3,4]. Therefore, optimization in brain CT, reducing radiation dose while preserving image quality and diagnostic performance, is a crucial issue in clinical radiology.

In CT, patients are exposed to X-rays generated by an X-ray tube. X-ray photons that pass through the patient are detected by the detector positioned opposite the X-ray tube and are used to reconstruct tomographic images. In larger patients, the proportion of X-rays that reach the detector decreases, which may increase image noise and impair diagnostic performance; thus, increased radiation exposure is required to keep image quality constant. Automatic exposure control modulates the strength of radiation exposure, primarily by modulating the tube current according to the degree of X-ray attenuation in the patient, and is used as a tool for optimization in CT [5,6,7]. Automatic exposure control has been shown to achieve appropriate dose modulation according to the head size in pediatric [8] and adult [9] brain CT.

Noise reduction reconstruction is another key technology for optimization in CT and includes model-based iterative reconstruction and hybrid iterative reconstruction (HIR) [10,11,12,13]. These techniques enable to decrease image noise compared to filtered backprojection (FBP), the conventional reconstruction method, and to provide diagnostic-quality images even with reduced radiation exposure. Although a long computation time prevents the widespread use of model-based iterative reconstruction, HIR offers fast processing and is widely adopted in clinical practice.

Recently, deep learning reconstruction (DLR) methods have been developed for noise reduction reconstruction based on artificial intelligence technology, and their application is expanding [14,15]. HIR affects image texture and may create artificial image features, termed as plastic, blotchy, or oil-painting appearance, which is considered a significant drawback of HIR [11,16,17,18]. Many reports have demonstrated the superiority of DLR over HIR in CT [14,19], including brain CT [20,21,22,23,24,25,26,27].

The standard deviation (SD) of the Hounsfield unit is commonly used as an index of noise magnitude in CT images reconstructed using FBP, and the signal-to-noise ratio and contrast-to-noise ratio are calculated using the SD as the indicator of image noise. Although these metrics do not fully represent noise properties in non-FBP images [11,28], they are often used for quantitative comparisons between brain CT images reconstructed using HIR and DLR [20,21,22,23,24,25,26,27,29]. A lower SD is regarded as one of the proofs showing the superiority of DLR over HIR [20,21,22,25,27]. Notably, the degree of noise reduction can be adjusted in HIR and DLR, according to the operator’s choice of reconstruction setting. In previous clinical studies on brain CT, the influence of the level selection was not evaluated extensively, and the number of the reconstruction levels studied was one for both HIR and DLR [20,21,22,23,25], two for HIR and one for DLR [26], or one for HIR and three for DLR [24,27,29]. Although a lower SD was shown for DLR than HIR, using a higher level of HIR should allow further noise reduction despite possibly compromising diagnostic acceptability due to the alterations of image texture. When comparing HIR and DLR, the effects of the level selection should be considered. Additionally, a previous study demonstrated that decreasing slice thickness increased noise in the same manner for HIR and FBP, but the noise increase was less for DLR [30], indicating the need to compare HIR and DLR for each slice thickness. In previous brain CT studies comparing HIR and DLR, thin-slice images of 0.5 [22,25] or 0.625 mm [23,24,26] thickness were evaluated, suggesting the potential of thin-slice brain CT imaging. However, the slice thickness examined was one in most studies, whereas 5 and 0.625 mm images were assessed in one study [26].

Before implementing DLR in our clinical practice, we imaged a 16 cm dosimetry phantom and the head of an anthropomorphic phantom to simulate brain CT. To determine optimal reconstruction settings, we reconstructed images using various levels of DLR and HIR and examined the degree of noise reduction relative to slice thickness and tube current. Moreover, we reconstructed brain CT images in patients using various levels of DLR and HIR to evaluate noise reduction and visual image quality. The primary aim of this study was to investigate the noise reduction properties of DLR and HIR, focusing on reconstruction parameter selection.

## 2. Materials and Methods

### 2.1. Instruments and Imaging Parameters

CT images of phantoms and patients were acquired in the axial mode on a 64-detector-row CT scanner (Revolution Frontier; GE Healthcare, Milwaukee, WI, USA). The scan parameter included a tube voltage of 120 kV, a rotation time of 0.5 s (phantom imaging) or 1.0 s (patient imaging), and a beam width of 20 mm. The tube current settings are presented in subsequent sections. The slice thickness was 5, 2.5, 1.25, and 0.625 mm. A 16 cm dosimetry phantom and an anthropomorphic whole-body phantom (PBU-60; Kyoto Kagaku, Kyoto, Japan) were used in phantom imaging. The 16 cm dosimetry phantom is designed to simulate brain CT. The head portion of the anthropomorphic was used as a head phantom.

### 2.2. Various Levels of Noise Reduction in Phantom Imaging

CT images of the 16 cm dosimetry phantom and head phantom were obtained using various levels of noise reduction reconstruction methods. The tube current was fixed at 320 mA. Image reconstruction was performed using FBP, DLR (TrueFidelity; GE Healthcare), and HIR (adaptive statistical iterative reconstruction-V, ASiR-V; GE Healthcare). The levels of DLR used were low (DLR-L), medium (DLR-M), and high (DLR-H). The blending percentages of HIR used were from 10% (HIR10) to 100% (HIR100) with 10% increments. Higher blending percentages lead to greater noise reduction.

**Figure 1:**
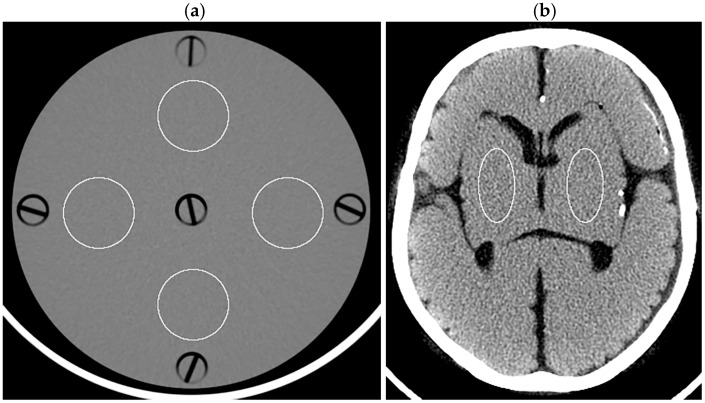
ROIs displayed on the 5 mm thick FBP images of the dosimetry phantom (**a**) and head phantom (**b**).

In analyzing the dosimetry phantom images, four circular regions of interest (ROIs, 766.6 mm^2^ each) were placed on a 5 mm thick image reconstructed with FBP and then applied to other images using ImageJ software (version 1.54g; National Institutes of Health, Bethesda, MD, USA) (Figure 1a). The SD of the Hounsfield unit was obtained for each ROI, and the mean SD across the four ROIs was defined as the image noise. For the head phantom, elliptical ROIs (527.4 mm^2^ each) representing the right and left lenticular nuclei were placed (Figure 1b). The SD was obtained for each ROI, and the right and left values were averaged to determine the image noise. Noise reduction ratios for various levels of DLR and HIR were calculated using the image noise for the FBP image of the corresponding thickness as the reference as follows:

noise reduction ratio = (N_FBP_ − N_HIR_ or N_DLR_)/N_FBP_ × 100

where N_FBP_ is the image noise for the FBP image, and N_HIR_ and N_DLR_ are those for the corresponding HIR and DLR images, respectively.

### 2.3. Various Tube Currents in Phantom Imaging

The 16 cm dosimetry phantom and head phantom were imaged at various tube currents (10, 20, 40, 80, 160, 320, and 640 mA). Images were reconstructed using FBP, DLR-M, and HIR50. Noise reduction ratios were determined in the same manner as in the analysis for various levels of noise reduction reconstruction.

### 2.4. Image Noise in Patient Imaging

Image noises in CT images created using various levels of noise reduction reconstruction were assessed in patient imaging. Eleven patients (seven men and four women), aged 54.1 ± 10.0 years (mean ± SD), who underwent plain CT of the brain for clinical indications and had normal findings, were included in the study. Image reconstruction was performed using various methods to reconsider our routine reconstruction protocols following the installation of the CT scanner with DLR capability. The study protocol was approved by the Kitasato University Medical Ethics Organization (B23-167), and the need for informed consent was waived.

**Figure 2:**
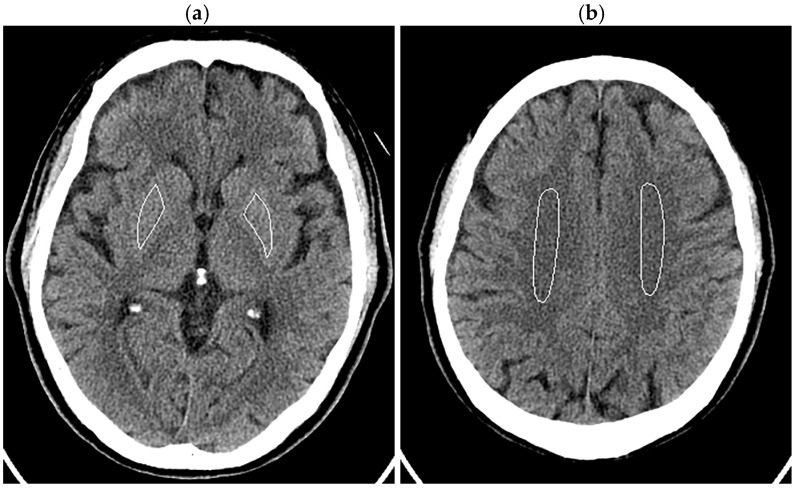
ROIs in the lenticular nuclei (**a**) and centrum semiovales (**b**) displayed on the 5 mm thick FBP images of the head of a 70-year-old male patient.

The patient’s head was imaged according to our routine clinical protocol. The tube current was modulated using inbuilt automatic exposure control software (Auto mA and Smart mA) with a noise index of 3.6, maximum mA of 350 mA, and minimum mA of 50 mA. Organ dose modulation was applied over the orbit to reduce radiation dose to the eye lens. This function selectively reduces radiation exposure from the anterior direction and does not change posterior exposure [31,32]. Image reconstruction was performed using FBP, three levels of DLR, and ten levels of HIR, similar to the phantom experiments. In image analysis, ROIs were manually drawn in bilateral lenticular nuclei (right 166.5 ± 26.6 mm^2^, left 158.5 ± 29.3 mm^2^), avoiding calcifications in the globus pallidus, and centrum semiovales (right 449.6 ± 136.0 mm^2^, left 432.6 ± 98.5 mm^2^) (Figure 2). The SD was obtained for each ROI, and the mean of the right and left values was defined as the image noises for the lenticular nucleus and centrum semiovale. Noise reduction ratios were calculated using the image noise for the FBP images as the reference.

### 2.5. Visual Assessment of Patient Images

The qualities of brain CT images in the 11 patients were evaluated visually and independently by two board-certified diagnostic radiologists. The overall qualities of the DLR-M images and two sets of HIR images were compared for each slice thickness. The mean noise reduction ratio across the 11 patients increased with increasing blending percentage for HIR. Two HIR image sets with consecutive blending percentages were selected for evaluation so that the mean noise reduction ratio in the lenticular nucleus for the DLR-M image set was between those for the two HIR image sets. These selected HIR images with lower and higher blending percentages were termed HIR-L and HIR-H images, respectively. Two of the three image sets (DLR-M, HIR-L, and HIR-H sets) were displayed randomly on the left and right panels of the picture archiving and communication system (PACS) viewer. The observer compared overall image quality, considering the recognizability of normal anatomical structures, homogeneity within each structure, the visual noise level, and the sharpness of the border of each structure. This instruction was given to the observer in writing, without sample images or training sessions. The observers recorded their judgments using the following options: the left set is superior, equal, and the right set is superior. All slices of each image set were used for comparison. The center and width of the display window were set at 40 and 80 Hounsfield unit, respectively. Pairwise comparisons were made for three combinations (DLR-M vs. HIR-L, DLR-M vs. HIR-H, and HIR-L vs. HIR-H), with the observers blinded to the reconstruction method used.

### 2.6. Statistical Analysis

The results of visual assessment in patient imaging were examined using the exact binomial test with Bonferroni correction with R software (version 4.2.1, R Foundation for Statistical Computing, Vienna, Austria). A *p* value less than 0.05 was deemed statistically significant.

## 3. Results

### 3.1. Various Levels of Noise Reduction in Phantom Imaging

The 16 cm dosimetry phantom and head phantom were imaged, and CT images of different slice thicknesses were reconstructed using FBP and various levels of DLR and HIR to determine noise reduction ratios. The image noise increased with decreasing thickness when using FBP (Table 1).

**Figure 3:**
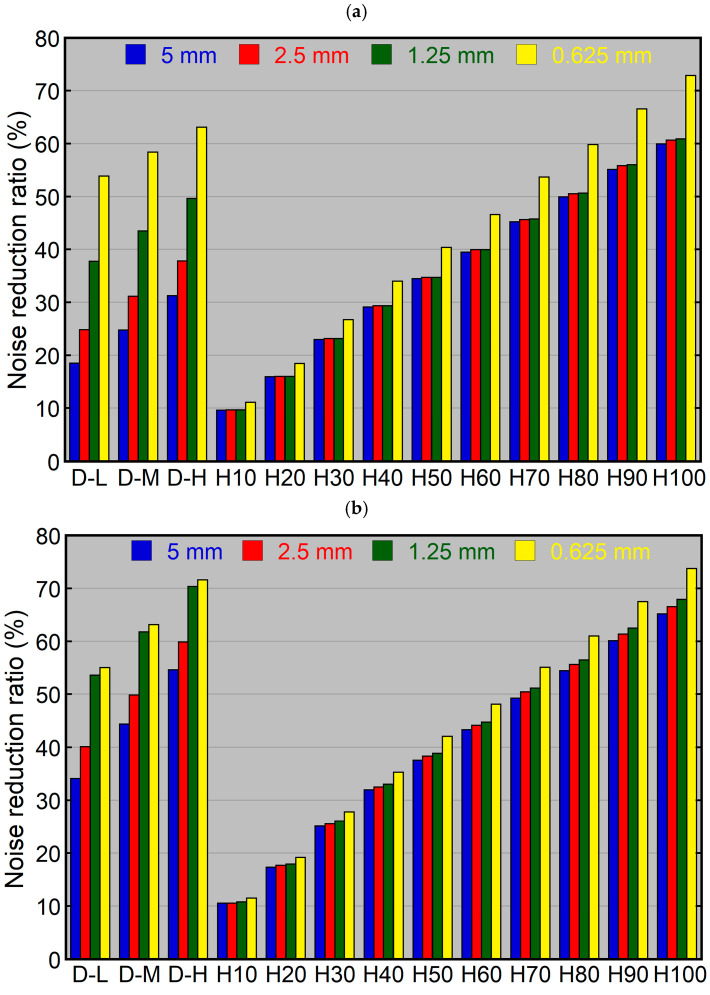
Noise reduction ratios using various levels of noise reduction reconstruction for the dosimetry phantom (**a**) and head phantom (**b**). The 5, 2.5, 1.25, and 0.625 mm thick images were reconstructed using various levels of DLR and HIR. D and H indicate DLR and HIR, respectively.

Using HIR, the noise reduction ratio increased with increasing blending percentages for both the dosimetry phantom (Figure 3a) and head phantom (Figure 3b). At a given blending percentage, the noise reduction ratio was similar among 5, 2.5, and 1.25 mm thick images and higher for 0.625 mm thick images. The difference in noise reduction ratios between 5 and 0.625 mm thick images ranged from 1.5% (HIR10) to 12.9% (HIR100) for the dosimetry phantom and from 1.0% (HIR10) to 8.6% (HIR100) for the head phantom. Using identical reconstruction parameters (same blending percentage and slice thickness), the noise reduction ratio was mildly higher for the head phantom than the dosimetry phantom, with a maximum difference of 7.0% (HIR100, 1.25 mm).

Using DLR, the noise reduction ratio was highest for DLR-H, followed by DLR-M and DLR-L (Figure 3). It differed largely depending on slice thickness, showing larger thickness dependence than HIR. The noise reduction ratio increased with decreasing slice thickness, and this tendency was more pronounced for the dosimetry phantom than for the head phantom. Using the same level of DLR, the difference in noise reduction ratio between the 5 and 0.625 mm thick images was 35.3%, 33.6%, and 31.8% for DLR-L, DLR-M, and DLR-H, respectively, in the dosimetry phantom, and was 20.9%, 18.8%, and 17.1% for DLR-L, DLR-M, and DLR-H, respectively, in the head phantom. Using identical reconstruction parameters, the noise reduction ratios for the 5, 2.5, and 1.25 mm thick images were higher for the head phantom than for the dosimetry phantom. This phantom-dependent difference was more pronounced for DLR than for HIR, ranging from 15.3% (DLR-L, 2.5 mm) to 23.3% (DLR-H, 5 mm). It was relatively small for the 0.625 mm thick images.

The blending percentage of HIR that resulted in a noise reduction ratio closest to that of DLR-M varied depending on the slice thickness and phantom. It was 30%, 40%, 70%, and 80% for the 5, 2.5, 1.25, and 0.625 mm thick images, respectively, using the dosimetry phantom, and was 60%, 70%, 90%, and 80%, respectively, using the head phantom.

### 3.2. Various Tube Currents in Phantom Imaging

The 16 cm dosimetry phantom and head phantom were imaged using various tube currents. Images of different slice thicknesses were reconstructed using FBP, DLR-M, and HIR50, and the effects of the tube current on the noise reduction ratio were evaluated. The image noise decreased with increasing tube current when using FBP (Table 2).

**Figure 4:**
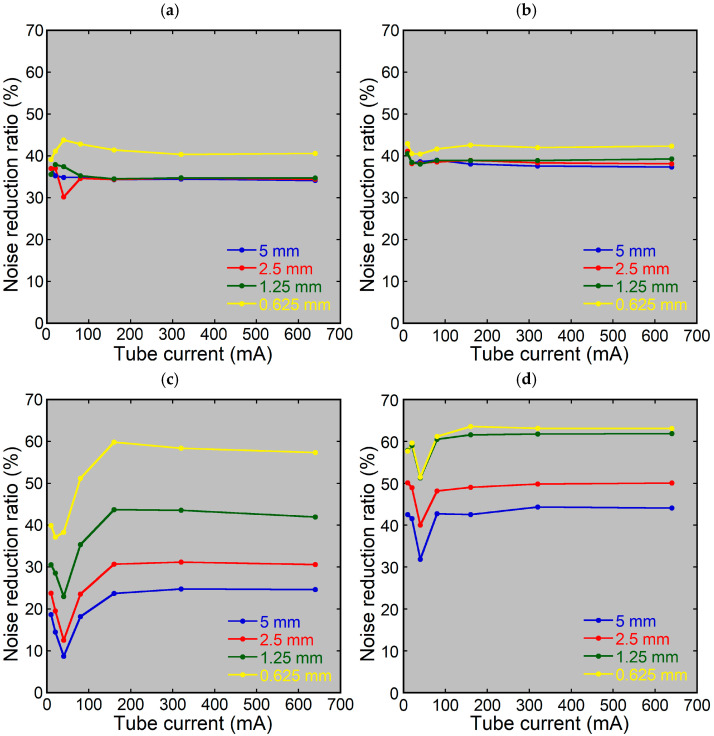
Noise reduction ratios using various tube currents. The 5, 2.5, 1.25, and 0.625 mm thick images of the dosimetry phantom (**a**,**c**) and head phantom (**b**,**d**) were reconstructed using HIR50 (**a**,**b**) or DLR-M (**c**,**d**).

Using HIR, the tube current had minimal effect on the noise reduction ratio, regardless of the phantom or slice thickness (Figure 4a,b).

Using DLR, the noise reduction ratio for the dosimetry phantom was low at low tube currents, especially at 40 mA, regardless of the slice thickness (Figure 4c). The ratio remained nearly constant at tube currents of 160 mA or higher. For the head phantom, the effects of the tube current were less pronounced; the noise reduction ratio was low at 40 mA, regardless of the slice thickness, and was similar at the other tube currents (Figure 4d).

### 3.3. Image Noise in Patient Imaging

In brain CT of the patients, the volume CT dose index (CTDIvol) and dose-length product (DLP) were 41.3 ± 6.2 mGy and 599.2 ± 116.9 mGy∙cm, respectively. They were lower than the Japanese diagnostic reference levels (CTDIvol, 77 mGy; DLP, 1350 mGy∙cm) [33]. The tube currents at the levels of the lenticular nucleus and centrum semiovale were 235.2 ± 43.7 mA and 229.5 ± 34.4 mA, respectively.

Brain CT images of different slice thicknesses were reconstructed using FBP and various levels of DLR and HIR, and the noise reduction ratios were evaluated. The image noise increased with decreasing thickness when using FBP (Table 3).

**Figure 5:**
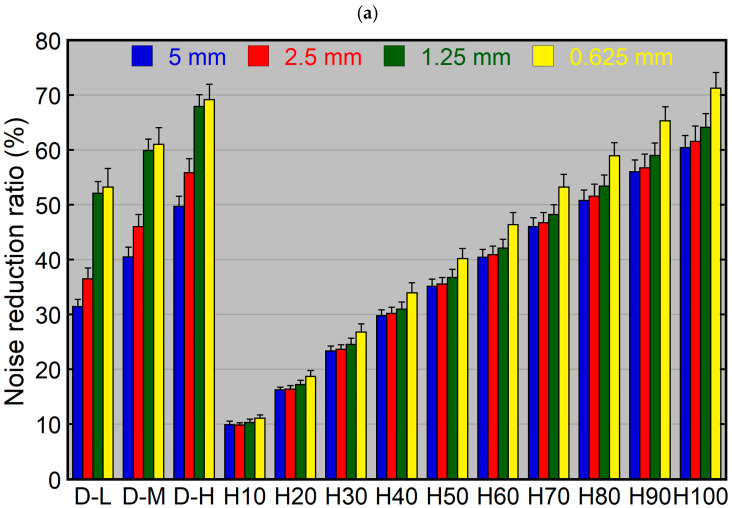

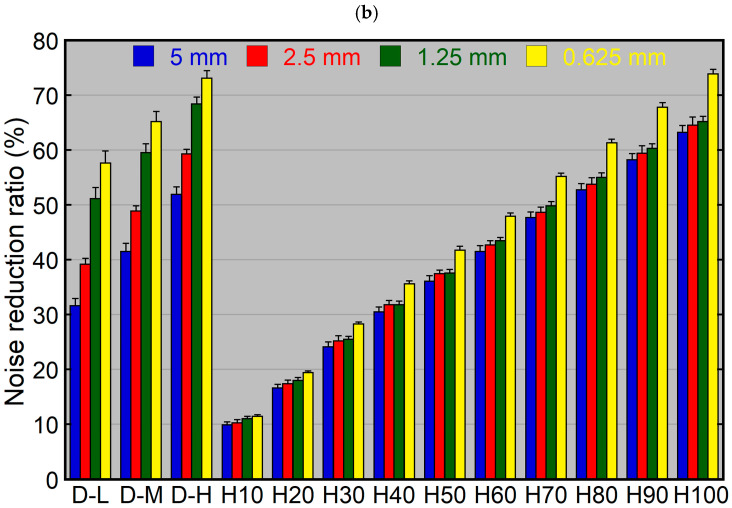
Noise reduction ratios for the lenticular nucleus (**a**) and centrum semiovale (**b**) in patient imaging. The 5, 2.5, 1.25, and 0.625 mm thick images were reconstructed using various levels of DLR and HIR. D and H indicate DLR and HIR, respectively. The error bar indicates the SD.

The difference in the noise reduction ratio between the lenticular nucleus and centrum semiovale was minimal using both HIR and DLR (Figure 5). The noise reduction ratio for the centrum semiovale minus that for the lenticular nucleus using the same reconstruction parameters ranged from 0.0% (HIR10, 5 mm) to 3.0% (HIR100, 2.5 mm) for HIR and from −1.0% (DLR-L, 1.25 mm) to 4.4% (DLR-L, 0.625 mm) for DLR.

Using HIR, the noise reduction ratio for the lenticular nucleus did not differ substantially from those for the dosimetry phantom and head phantom. The noise reduction ratio for the lenticular nucleus minus that for the phantom using the same reconstruction parameters ranged from −1.6% (HIR100, 0.625 mm) to 3.2% (HIR100, 1.25 mm) for the dosimetry phantom and from −5.0% (HIR100, 2.5 mm) to −0.4% (HIR10, 0.625 mm) for the head phantom.

Using DLR, the noise reduction ratios on the 5, 2.5, and 1.25 mm thick images were higher for the lenticular nucleus than for the dosimetry phantom using the same reconstruction parameters, and the difference ranged from 11.7% (DLR-L, 2.5 mm) to 18.5% (DLR-H, 5 mm). The difference was small on the 0.625 mm thick images. The noise reduction ratio was slightly lower for the lenticular nucleus than for the head phantom using the same reconstruction parameters, with the difference ranging from 1.5% (DLR-L, 1.25 mm) to 4.8% (DLR-H, 5 mm).

### 3.4. Visual Assessment of Patient Images

Based on the mean noise reduction ratio for the lenticular nucleus in the 11 patients, the following HIR images were selected for visual comparison with DLR-M images: 5 mm thick HIR60 and HIR70 images, 2.5 mm thick HIR60 and HIR70 images, 1.25 mm thick HIR90 and HIR100 images, and 0.625 mm thick HIR80 and HIR90 images. For example, HIR60 and HIR70 were designated as HIR-L and HIR-H, respectively, for 5 mm thick images.

**Figure 6:**
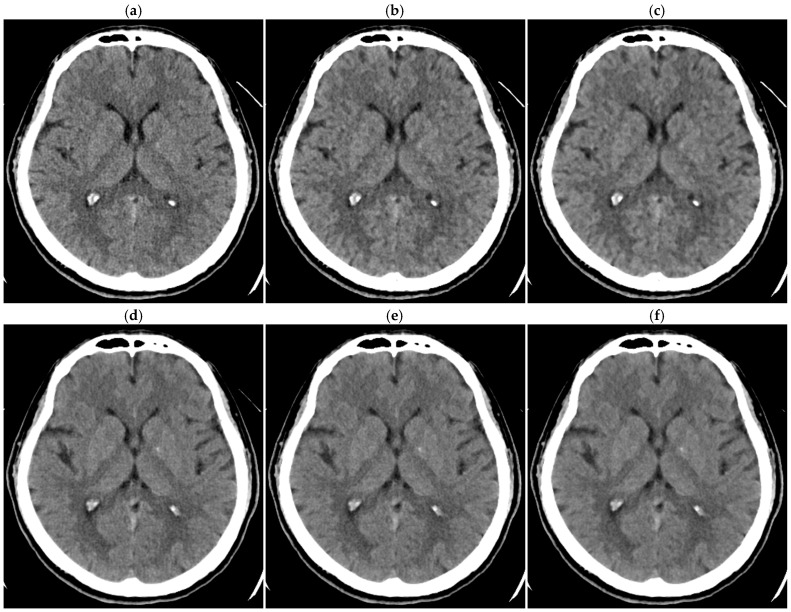
Brain CT images of 1.25 mm thickness ((**a**) DLR-M; (**b**) HIR-L; (**c**) HIR-H) and 5 mm thickness ((**d**) DLR-M; (**e**) HIR-L; (**f**) HIR-H) in a 38-year-old male patient.

For 1.25 mm thick images, both observers judged DLR-M to be superior to HIR-L and HIR-H in all patients (*p* < 0.01; Table 4, Figure 6). For 0.625 mm thick images, one observer judged DLR-M to be significantly superior to HIR-L and HIR-H (*p* < 0.05 for both). The other observer judged DLR-M to be significantly superior to HIR-L (*p* < 0.05); however, the superiority of DLR-M over HIR-H was not statistically significant. For 5 mm thick images, both observers’ judgments were similar between DLR-M and HIR-L, indicating selecting HIR-L allows HIR to provide image quality comparable to DLR-M. For 2.5 mm thick images, one observer tended to judge DLR-M as superior to HIR; however, the difference between DLR-M and HIR-H was not statistically significant. The other observers’ judgment was almost identical between DLR-M and HIR-L. Overall, significant differences were not indicated between DLR-M and HIR for 2.5 mm thick images.

## 4. Discussion

DLR has been reported to produce less noisy brain CT images compared to HIR [20,21,22,25,27]. However, the degree of noise reduction can be adjusted in both HIR and DLR depending on the selection of the noise reduction level. Therefore, the results of image noise comparison should vary according to the selection. In this study, we reconstructed brain CT images using various levels of DLR and HIR and evaluated noise reduction effects. The SD in the ROI was used as the indicator of image noise. Although the SD and SD-based metrics (signal-to-noise ratio and contrast-to-noise ratio) do not fully represent noise properties in non-FBP images [11,28], they have often been used in previous brain CT studies comparing HIR and DLR [20,21,22,23,24,25,26,27,29], as well as for quality control in routine clinical practice. Understanding how reconstruction conditions affect the SD can provide valuable insights.

Although the effects of the slice thickness on noise reduction in the dosimetry phantom, head phantom, and patients were small using HIR, the noise reduction ratio increased significantly with decreasing slice thickness using DLR. The noise in FBP images increases with decreasing thickness, and DLR mitigates this increase, which is consistent with a previous study [30]. Thin-slice DLR images have been reported to outperform both thin- and thick-slice HIR images [26,34,35,36], which may be partly due to the predominant noise reduction in thin-slice images using DLR. As a result of the dependence of the noise reduction ratio on the slice thickness for DLR, the blending percentage of HIR that yielded a noise reduction ratio closest to that of DLR-M varied depending on the slice thickness. When introducing DLR into clinical practice, the reconstruction conditions should be determined for each slice thickness taking into account the dependence of noise reduction on the slice thickness.

Phantom experiments were conducted to assess the impact of the tube current on noise reduction. Using HIR, the noise reduction ratio for the dosimetry phantom and head phantom remained nearly unchanged regardless of the tube current. In contrast to HIR, the noise reduction ratio for the dosimetry phantom varied with the tube current using DLR. It was low at low tube currents and remained nearly constant at 160 mA or higher. For the head phantom, the effect of the tube current was less pronounced. A dip at 40 mA was observed in the relationship of the noise reduction ratio against the tube current for both the dosimetry phantom and head phantom. We repeated the experiments assessing the tube current dependence and obtained similar results. The influence of electronic noise increases at a lower tube current due to a decrease in true signal. On a GE scanner, low count correction is applied during image reconstruction to depress image noise deriving from electronic noise. The effect of this correction may be similar between FBP and HIR but different between FBP and DLR, presumably causing the curious changes in the noise reduction ratio at low currents. The tube current dependence for DLR was consistent across different slice thicknesses. Regarding the increased noise reduction ratio for thinner slices using DLR, thinner slices are noisier, and it may be hypothesized that the thickness dependence is due to the dependence of the noise reduction ratio on the noise magnitude, with noisier images showing greater noise reduction. However, the results of the experiments using various tube currents contradict this hypothesis. A reduction in the tube current increased image noise but did not increase the noise reduction ratio. The findings suggest that the slice thickness directly affects the noise reduction ratio, independent of the noise level.

Using HIR, differences in the noise reduction ratio were small among the dosimetry phantom, head phantom, and patients. This suggests that noise reduction in patient imaging can be predicted using a phantom with simple geometry. ASiR-V, HIR on a GE scanner, allows the level selection from ten options, which is a merit of this technique. The noise reduction ratio increased gradually with the elevation of the level. Detailed optimization of the level is advisable when using ASiR-V. In contrast to HIR, the noise reduction ratio using DLR varied significantly between the dosimetry phantom and head phantom. Additionally, the effects of the slice thickness and tube current on the noise reduction ratio differed between the two phantoms. Notably, the degree of noise reduction using DLR depends on the imaging object. However, differences in the noise reduction ratio between the head phantom and patients were small, indicating that the degree of noise reduction in patient brain CT can be reasonably predicted using a head phantom.

The overall quality of clinical brain CT images with normal findings was compared between HIR and DLR. The selection of the noise reduction level for each reconstruction method should influence the results of such comparison. In this study, DLR-M was selected for visual assessment, and the blending percentages of HIR were selected to achieve noise reduction similar to DLR-M, based on the SD. For preliminary assessment, only two observers and a small number of normal CT examinations were employed. Considering the small scale of the assessment, a pairwise comparison of overall quality was conducted instead of grading each item (noise, sharpness, contrast, diagnostic acceptability, etc.) for each image set. We expected better reproducibility for pairwise comparison than grading. Although visual quality was comparable for thick-slice images (5 and 2.5 mm), DLR tended to be superior for thin-slice images (1.25 and 0.625 mm). For thin-slice images, the noise reduction was large using DLR, and a high blending percentage was required for HIR to achieve a similar degree of noise reduction. A high blending percentage resulted in a so-called plastic appearance and degraded visual image quality, despite similar SD. If the image noise is acceptable, decreasing slice thickness is expected to improve the clarity of the normal structures and lesions due to depressing the partial volume effect. DLR may be superior to HIR for thin-slice imaging, as indicated in previous studies [26,34,35,36], potentially improving diagnostic performance.

There are limitations to this study. First, only one CT scanner with built-in HIR and DLR was used. The characteristics of HIR and DLR may differ depending on the manufacturer; reconstruction methods provided by other manufacturers should be studied in the future. Second, the SD was used as a quantitative indicator of noise. A more detailed assessment considering alterations in image texture would be beneficial. Third, in the visual assessment of patient images, only overall image quality was assessed by only two observers on a small number of patients with normal findings. A larger-scale evaluation of lesion detectability and conspicuity remains to be carried out. Finally, although HIR image sets with SDs similar to DLR-M image sets were selected for visual comparison with DLR-M images, the best HIR and DLR image sets may be selected visually among various HIR and DLR image sets, respectively, for future comparison between HIR and DLR.

## 5. Conclusions

Noise reduction properties in brain CT were evaluated using various levels of DLR and HIR. In HIR, the degree of noise reduction was primarily determined by the blending percentage, with limited influence of the slice thickness, tube current, or imaging object. DLR exhibited more complex behavior, and the degree of noise reduction was affected not only by the level of DLR but also by the slice thickness, tube current, and imaging object. The head phantom appeared to be better than the dosimetry phantom in predicting noise reduction in patients. These characteristics of DLR should be considered in its clinical use and comparing it to HIR. DLR may be particularly beneficial for thin-slice imaging.

## Figures and Tables

**Table 1:** FBP image noise in phantom experiments for various levels of noise reduction reconstruction.

Phantom	Image Noise			
	5 mm	2.5 mm	1.25 mm	0.625 mm
Dosimetry	3.98	5.60	7.88	11.25
Head	5.41	7.39	11.34	12.39

**Table 2:** FBP image noise in phantom experiments using various tube currents.

Tube Current (mA)	Image Noise							
	Dosimetry Phantom				Head Phantom			
	5 mm	2.5 mm	1.25 mm	0.625 mm	5 mm	2.5 mm	1.25 mm	0.625 mm
10	23.69	34.59	47.24	66.52	31.21	42.18	56.72	57.83
20	16.12	23.49	34.10	47.70	21.27	28.32	38.51	39.91
40	11.16	16.10	23.26	34.45	15.73	20.73	28.82	30.19
80	7.73	11.05	15.82	23.43	10.71	14.63	20.81	21.93
160	5.57	7.82	11.04	16.32	7.26	10.10	15.44	16.98
320	3.98	5.60	7.88	11.25	5.41	7.39	11.34	12.39
640	2.82	3.89	5.37	7.78	3.73	5.12	7.92	8.78

**Table 3:** FBP image noise in patient imaging.

Region	Image Noise			
	5 mm	2.5 mm	1.25 mm	0.625 mm
Lenticular Nucleus	4.71 ± 0.29	6.56 ± 0.40	9.57 ± 0.57	10.41 ± 0.70
Centrum Semiovale	4.07 ± 0.16	5.80 ± 0.19	8.50 ± 0.39	10.47 ± 0.71

Values are mean ± SD.

**Table 4:** Visual assessment of patient images.

Comparison	Number of Judgments							
	5 mm		2.5 mm		1.25 mm		0.625 mm	
	Ob #1	Ob #2	Ob #1	Ob #2	Ob #1	Ob #2	Ob #1	Ob #2
DLR-M > HIR-L	1	1	8 ^b^	1	11 ^a^	11 ^a^	9 ^b^	7 ^b^
DLR-M = HIR-L	10	10	3	10	0	0	2	4
DLR-M < HIR-L	0	0	0	0	0	0	0	0
DLR-M > HIR-H	6	4	6	5	11 ^a^	11 ^a^	6	8 ^b^
DLR-M = HIR-H	5	7	5	5	0	0	5	3
DLR-M < HIR-H	0	0	0	1	0	0	0	0
HIR-L > HIR-H	5	2	2	3	8 ^b^	9 ^b^	0	7 ^b^
HIR-L = HIR-H	5	8	7	8	3	2	5	4
HIR-L < HIR-H	1	1	2	0	0	0	6	0

Ob #1, observer 1; Ob #2, observer 2; ^a^, *p* < 0.01; ^b^, *p* < 0.05.

## Data Availability

The data are available upon reasonable request from the corresponding author.
